# IRF-1 expressed in the inner cell mass of the porcine early blastocyst enhances the pluripotency of induced pluripotent stem cells

**DOI:** 10.1186/s13287-020-01983-2

**Published:** 2020-11-27

**Authors:** Bingbo Shi, Dengfeng Gao, Liang Zhong, Minglei Zhi, Xiaogang Weng, Junjun Xu, Junhong Li, Xuguang Du, Yanli Xin, Jie Gao, Qianqian Zhu, Suying Cao, Zhonghua Liu, Jianyong Han

**Affiliations:** 1 State Key Laboratory for Agrobiotechnology, College of Biological Sciences, China Agricultural University, Beijing, 100193 China; 2 Hebei Provincial Key Laboratory of Basic Medicine for Diabetes, The Shijiazhuang Second Hospital, Shijiazhuang, 050051 Hebei China; 3 Key Laboratory of Animal Cellular and Genetics Engineering of Heilongjiang Province, College of Life Science, Northeast Agricultural University, Harbin, 150030 China; 4 Animal Science and Technology College, Beijing University of Agriculture, Beijing, 102206 China

**Keywords:** piPSCs, IRF-1, Pluripotency, JAK-STAT

## Abstract

**Background:**

Despite years of research, porcine-induced pluripotent stem cells (piPSCs) with germline chimeric capacity have not been established. Furthermore, the key transcription factors (TFs) defining the naïve state in piPSCs also remain elusive, even though TFs in the inner cell mass (ICM) are believed to be key molecular determinants of naïve pluripotency. In this study, interferon regulatory factor 1 (IRF-1) was screened to express higher in ICM than trophectoderm (TE). But the impact of IRF-1 on maintenance of pluripotency in piPSCs was not determined.

**Methods:**

Transcriptome profiles of the early ICM were analyzed to determine highly interconnected TFs. Cells carrying these TFs’ reporter were used to as donor cells for somatic cell nuclear transfer to detect expression patterns in blastocysts. Next, IRF1-Flag was overexpressed in DOX-hLIF-2i piPSCs and AP staining, qRT-PCR, and RNA-seq were conducted to examine the effect of IRF-1 on pluripotency. Then, the expression of IRF-1 in DOX-hLIF-2i piPSCs was labeled by GFP and qRT-PCR was conducted to determine the difference between GFP-positive and GFP-negative cells. Next, ChIP-Seq was conducted to identify genes target by IRF-1. Treatment with IL7 in wild-type piPSCs and STAT3 phosphorylation inhibitor in IRF-1 overexpressing piPSCs was conducted to confirm the roles of JAK-STAT3 signaling pathway in IRF-1’s regulation of pluripotency. Moreover, during reprogramming, IRF-1 was overexpressed and knocked down to determine the change of reprogramming efficiency.

**Results:**

IRF-1 was screened to be expressed higher in porcine ICM than TE of d6~7 SCNT blastocysts. First, overexpression of IRF-1 in the piPSCs was observed to promote the morphology, AP staining, and expression profiles of pluripotency genes as would be expected when cells approach the naïve state. Genes, KEGG pathways, and GO terms related to the process of differentiation were also downregulated. Next, in the wild-type piPSCs, high-level fluorescence activated by the IRF-1 promoter was associated with higher expression of naïve related genes in piPSCs. Analysis by ChIP-Seq indicated that genes related to the JAK-STAT pathway, and expression of IL7 and STAT3 were activated by IRF-1. The inhibitor of STAT3 phosphorylation was observed could revert the expression of primed genes in IRF-1 overexpressing cells, but the addition of IL7 in culture medium had no apparent change in the cell morphology, AP staining results, or expression of pluripotency related genes. In addition, knockdown of IRF-1 during reprogramming appeared to reduce reprogramming efficiency, whereas overexpression exerted the converse effect.

**Conclusion:**

The IRF-1 expressed in the ICM of pigs’ early blastocyst enhances the pluripotency of piPSCs, in part through promoting the JAK-STAT pathway.

## Background

Due to their high degree of genetic, physiologic, and anatomic similarities with humans, pigs are excellent models of human diseases and are suitable xenograft donors [1, 2]. Porcine pluripotent stem cells, including induced pluripotent cells (piPSCs) and porcine embryonic stem cells (pESCs) exhibit high quality with respect to colony formation, making complicated genetic manipulations of medical utility of relatively easy in this model system [3]. Furthermore, authentic naïve porcine pluripotent stem cells were expected to be used for the generation of chimeric fetuses or the production of functional germ stem cells or gametes, which has the potential to accelerate breeding [4, 5]. Compared with pESCs derived from porcine blastocysts, piPSCs were induced from somatic cells by reprogramming with pluripotency-associated transcription factors, which have the advantage of being readily available. Additionally, establishing iPSCs from rarer subspecies aids in the conservation of germplasm resources [6]. However, piPSCs that have germline chimeric capacity have not been thoroughly characterized.

Naïve and primed are two concepts proposed for murine pluripotent stem cells [7]. These proposed states have been developed into two common states in human, mouse, and monkey pluripotent stem cells [8, 9]. Mouse cells in the naïve state were isolated from the early epiblasts of mature blastocysts, while cells in the primed state were isolated from epiblasts after implantation. Between these two states, naïve cells are at an earlier developmental stage, have better pluripotency, allowing them to enter the embryonic development process to generate chimeric fetuses [7]. Primed cells are in a state of imminent differentiation, which have the advantage of being able to differentiate into other cell types. Because most of the reported piPSCs were not silent for exogenous genes and endogenous pluripotency genes are not activated, these cells are characterized as being either naïve-like or primed-like. The LIF-dependent naïve-like piPSCs exhibit similar morphological characteristics and gene expression patterns as mouse embryonic stem cells (ESCs), which have a domed morphology [10–15]. The primed-like piPSCs are bFGF-dependent and exhibit the similar flattened morphology as human primed ESCs and mouse epiblast stem cells [10–12, 15–18]. Neither naïve-like nor primed-like piPSCs have normal expression of marker genes associated with the naïve state. Recently, expanded potential porcine pluripotent stem cells have been established, which could form viable chimeras. However, the chimeric rate failed to exceed 1.7%, and the naïve marker genes TBX3 and NR5A2 were only expressed at low levels [19]. Therefore, the core regulatory factors of the naïve state of piPSCs require examination.

During the early blastocyst stage in mice, humans, and nonhuman primates, the inner cell mass (ICM) or preimplantation epiblast cells are used exclusively to derive naïve ESCs [20–25], as these cells possess transcriptomic features of naïve pluripotency [20, 26–28]. Mouse naïve ESCs derived from preimplantation epiblasts (EPI) [21, 22] exhibit molecular traits consistent with the mid-blastocyst-stage ICM, whereas E3.5d mouse embryos expresses the naïve marker genes NANOG, TBX3, TFCP2L1, and KLFs [7, 27]. In humans and nonhuman primates, naïve ESCs could be established from cells of the ICM expressing characteristic marker genes, such as KLF17 [20, 24, 25, 29]. In pigs, it has been hypothesized that naïve stem cells might rely on the regulation of special transcription factors in the ICM, in which the naïve signature is only expressed in a short window [30]. In this study, the special transcription factors of porcine ICM were analyzed. As a result of the preliminary analysis, interferon regulatory factor 1 (IRF-1) was screened for ICM-specific expression.

The IRF-1 protein is a member of the IRF transcription factor family, a well-known family of regulators of the type I interferon system [31]. It was the first of the IRFs to be discovered and was observed to be a transcriptional regulator of the human IFN-β, IFN-γ, and MHC class I genes [32]. In mouse cellular immune responses, IRF-1 was observed to translocate to the nucleus, leading to induction of a specific subset of genes, including IFN-β, inducible NO synthase, and IL-12p35 [33]. In addition to innate immune responses, it has also been reported that IRF-1 functions in the differentiation of T-helper cells and exhibits anticancer activities [34–37]. With respect to pluripotency, as was previously described, single cell RNA-seq analysis of cells during reprogramming also indicated that a group of primary immune genes including IRF-1 were specifically expressed from the middle to the late stage of reprogramming [38]. The TF activity of the IRF family was enriched in the prior iPSCs stage as well [38]. However, the impact of IRF-1 on the maintenance of pluripotency in pluripotent stem cells has received little attention in mice, humans, or pigs alike.

In the present study, specific fluorescence activated by the IRF-1 promoter in porcine ICM was detected. These observations support IRF-1 might regulate the pluripotency of piPSCs. Overexpression of IRF-1 in piPSCs resulted in increased pluripotency as well as an inhibition of genes and pathways related to differentiation. ChIP-seq was performed to determine that IRF-1 could bind the IL7 and STAT3 genes, which have been associated with JAK-STAT signaling pathway. In addition, knockdown and overexpression of IRF-1 during reprogramming suggested that IRF-1 is a positive regulator of reprogramming. For the first time, our findings illustrate the ability of IRF-1 to enhance the pluripotency of piPSCs.

## Methods

### Vector construction

Reporter plasmids using the Sleeping Beauty transposon system for BCL3, IRF-1, DNMT1, GTF3C1, MCM4, NCOA1, SOX2, STAT3, and TCF3 were constructed. Briefly, the backbone PT2-GFP-mCherry was constructed by introducing the EF1-GFP-Puro cassette to replace the Puro cassette in PT2-Puro-mCherry [39]. PT2-Puro-mCherry was linearized with EcoR I and Hind III and then assembled with the EF1-GFP-Puro cassette amplified by PCR from PB-CMV-MCS-EF1α-GreenPuro (PB513B, System Biosciences) using the NEBuilder®HiFi DNA Assembly Master Mix (NEW E2621L, ENGLAND BioLabs). The promoter fragments were then amplified from the DNA of porcine embryonic fibroblasts (PEFs), which were assembled into the linearized PT2-GFP-Puro-mCherry between the EcoR I and Bgl II restriction sites. The primers used in this process are presented in Table S1. All of the above reporter plasmids were transfected into porcine cells along with the transposase vector (pCMV (CAT)T7-SB100, Addgene #34879), which mediates random insertion of specific promoter driven mCherry cassettes and conferred constitutive GFP expression.

Reporter plasmids for the detection of heterogeneity of IRF1 in piPSCs were constructed. The IRF-1 promoter was cut from PT2-GFP-mCherry and inserted into the pT2/LTR7-GFP plasmid (Addgene #62541) between the EcoRI and AgeI. The NeoR/KanR cassette that confers resistance to G418 was amplified from pCDNA3.1 using the primers NeoR/KanR-F/R. The amplicon was assembled into the linear vector between the EcoRI and EcoRV sites, yielding the construct PT2-Neo-IRF1-GFP. Finally, the vector was transfected along with the transposase vector pCMV (CAT) T7-SB100, which mediated random insertions of the IRF-1 promoter driven GFP cassette.

For IRF1 and BCL3 overexpression studies, the pCAG-IRES-Puro expression vector was used as the backbone for delivery of the genes. The fragment of IRF1 with the C-terminal flag tag was amplified from the cDNA of PEFs using primers IRF1-flag-F/R and was then assembled into the MluI and PacI sites of pCAG-IRES-Puro to construct PB-CAG-IRF1-Flag. For BCL3 overexpression in piPSCs, the exon 1 of BCL3 was amplified from the cDNA of PEFs using primers BCL3-ex1F/R. Exons 2-8 of BCL3 were amplified from PEFs’ cDNA using primers BCL3-ex2-8 F/R. The resulting amplicons were assembled into the linearized pCAG-IRES-Puro backbone between the MluI and PacI sites, yielding PB-CAG-BCL3. Next, the retroviral packaging plasmid PMX-IRF1 was used for overexpression of IRF-1 during reprogramming was constructed by assembling the CDS of IRF1 with the PMX vector linearized by EcoRI and XhoI [40]. As a control, PMX-tdTomato was also constructed by assembling tdTomato CDS with the linearized PMX vector.

The plasmids for RNAi mediated knockdown of IRF1 were created as follows: primers for shRNA targeting IRF1 (presented in Table S1) were annealed and cloned into the pSuper-puro (VEC-PBS-0008, Oligoengine) vector between the Bgl II and Hind III restriction sites, and subsequently cloned into the pLVTHM vector (Addgene #12247). Then, pLVTHM-sh5 and sh6 vectors were used to package lentiviral vectors for IRF1 knockdown, while pLVTHM-luciferase was used as a negative control.

### Packaging of virus

Retrovirus vectors were packaged using PMX-pOKSM [41], PMX-IRF1, and PMX-tdTomato. Briefly, 12 μg of PMXs and 4 μg of pVSVG were transfected into GP2-293 cells cultured in T75 flask using the Lipo-2000 transfection reagent. The knockdown (PLVTH) and reprogramming (FUW-OSKM and FUW-M2rtTA [42]) plasmids were used to package lentivirus. Briefly, 10 μg lentivirus plasmids, 8 μg p8.91, and 6 μg pVSVG were transfected into 293-FT cells cultured in T75 flasks. Culture supernatants were harvested 48 h post-transfection, filtered through 0.45-μm sterile filters, concentrated using PEG8000 Virus Precipitation Solution (5×) overnight at 4 °C. The virus containing culture supernatants were pelleted and resuspended in 200 μl opti-MEM.

### Generation of PEFs carrying reporter system and SCNT for fluorescence detection in blastocyst

PEFs derived from porcine embryos at day 40 [41] were resuscitated and transfected with reporter plasmids for BCL3, IRF-1, DNMT1, GTF3C1, MCM4, NCOA1, SOX2, STAT3, and TCF3. Positive cells were obtained by selection with 1 μg/mL puromycin for more than 3 days and were cultured to confluence. Pig ovaries were acquired from a local slaughterhouse and transported in 0.9% saline at 35–38 °C. Cumulus oocyte complexes (COCs) were extracted from the oocytes with a 12-gauge needle, then washed and transferred into maturation medium. The COCs were cultured at 38.5 °C and 5% CO_2_ for 42–44 h and then digested using 0.1% (w/v) hyaluronidase (H4272, Sigma). Mature oocytes were collected and used as recipients for SCNT. Next, PEFs carrying the above-described reporter were digested into single cell suspensions. Cells exhibiting bright fluorescence were picked as donor cells under inverted fluorescence microscope. The SCNT procedure was performed as previously described [43]. Then, SCNT embryos were cultured in porcine zygote medium-3 to the blastocyst stage for fluorescence detection.

### Generation and identification for DOX-hLIF-2i piPSCs

The PEFs carrying OCT4-tdTomato reporter were obtained from Liangxue Lai’s laboratory [44]. These PEFs were transduced with FUW-OSKM and FUW-M2rtTA lentiviral vectors and then incubated for 7 days in DMEM (11960, Gibco) supplemented with 10% FBS (SE200-ES, VISTECH), 1% Glutamax (35050061, Gibco), and 1% penicillin/streptomycin (15140-122, Gibco). Next, these PEFs were seeded onto feeder cells at a density of 40,000 cells/well into 6-well plate. Two days later, cells were changed to pLIF+2i+DOX FBS-KOSR medium consisting of DMEM supplemented with 10%FBS, 10% KOSR (10828-028, KOSR), 1% non-essential amino acid (11140050, Gibco), 1% Glutamax, 1% penicillin/streptomycin, 0.1 mM β-mercaptoethanol (21985023, Gibco), 3 μM CHIR99021 (Selleck, S1263), 1 μM PD0325901 (Selleck, S1036), 2 μg/mL Doxycycline hyclate (24390-14-5, Sigma), and porcine LIF conditioned medium (pLIF) at 1:500. The pLIF conditioned medium was collected from culture supernatants of pLIF-expressing CHO cells, which were constructed by transfected pLIF-expressing vector into CHO-K1 cells. CHO-K1 cells (GDC018) were purchased from China Center For Type Culture Collection (Wuhan, China) and the pLIF-expressing vector was constructed as described previously [12]. After 10 days, dome-like colonies were picked, detached with TripleTM Express (12605036, Gibco), and dispersed on feeder cells in hLIF+pLIF+2i+DOX+ 15% FBS medium, in which 10% FBS and 10% KOSR were replaced by 15% FBS and 10 ng/ml human LIF (Millipore, LIF1005) was added. More than three colonies were passaged for the generation of stable cell lines. To obtain cell lines with better morphology, compact colonies with clear margins were picked from cells at Passage 8 and cultured in hLIF+pLIF+2i+DOX N2/B27 medium (50% (v/v) Neurobasal™Medium (21103-049, Gibco), 50% (v/v) DMEM/F12 (10565-018, Gibco), 1× N2 (17502-048, Gibco), 0.5× B27 (12587-010, Gibco)), 5% KOSR, 1% non-essential amino acid, 1% Glutamax and 1% penicillin/streptomycin, 0.1 mM β-mercaptoethanol, 2 μg/mL Doxycycline hyclate, 3 μM CHIR99021, 1 μM PD0325901, 10 ng/ml human LIF, and pLIF conditioned medium at 1:500. Then, 3 homogeneous stable cell lines were established, which were named DOX-hLIF-2i piPSCs. Pluripotency of DOX-hLIF-2i piPSCs was detected by in vitro differentiation, immunofluorescence, alkaline phosphatase (AP), and qRT-PCR.

### Overexpression of IRF-1 in DOX-hLIF-2i piPSCs

Overexpression of IRF1-Flag in DOX-hLIF-2i piPSCs was accomplished by transfecting the cells with PB-CAG-IRF1-Flag and PCAGPBase plasmids. The control cells were transfected with PB-CAG-Flag. After transfection, cells were obtained by selection with 0.5 μg/mL puromycin for 3 days. The positive cells were passaged and collected for RNA extraction and AP staining.

### Treatment with IL7 in WT piPSCs and STAT3 phosphorylation inhibitor in IRF-1 overexpressing piPSCs

To explore effect of IL7 on DOX-hLIF-2i piPSCs, cells were treated with 0 ng/ml, 10 ng/ml, and 25 ng/ml IL7 (200-07-2, PeproTech) for 4 days. Treated cells were then assayed by AP staining and qRT-PCR. To explore the role of the JAK-STAT3 signaling pathway in IRF-1 promotion pluripotency in piPSCs, IRF-1 overexpressing piPSCs were treated with 2.5 μM STAT3 phosphorylation inhibitor, Stattic (S7024, Selleck) for 3 d. Treated cells and control cells were detected by AP staining and qRT-PCR.

### Generation of IRF1-GFP reporter cells and flow cytometry analysis

To obtain piPSCs carrying the IRF1-GFP reporter, DOX-hLIF-2i piPSCs were transfected with PT2-Neo-IRF1-GFP and pCMV (CAT) T7-SB100. Cultures were subjected to 400 μg/ml G418 selection for 7 days. The fluorescence of GFP was analyzed using a MoFlo® High-Performance Cell Sorter (Beckman Coulter). Both GFP-positive and -negative cells were sorted and collected for culture. Owing to the low rate of GFP-positive cells, colonies of GFP-positive cells were picked and cultured for a second round of sorting. Then, the difference between GFP-positive and -negative cells were identified by AP staining and qRT-PCR.

### IRF-1 knockdown during reprogramming

PEFs derived from porcine embryos at day 40 [41] were resuscitated and infected with retrovirus PMX-pOSKM and lentivirus PLVTH to knockdown expression of IRF-1. These cells were seeded in 6-well plate at a density of ratio 40,000 cells/ well. The time seeding was recorded as day 0. After 24 h, the culture medium was switched to induction medium (hLIF+15% FBS) for reprogramming, in which DMEM supplemented with 15%FBS, 1% non-essential amino acid, 1% Glutamax, 1% penicillin/streptomycin, 0.1 mM β-mercaptoethanol, and 10 ng/ml recombinant human LIF were included. After 5 days, cells were switched to hLIF+15% FBS culture medium containing 3 μM CHIR99021 and 1 μM PD0325901 for another 15 days. AP staining was performed at day 20 of reprogramming. Numbers of AP-positive colonies were statistically analyzed for comparison.

### IRF-1 overexpression during reprogramming and generation of OKSMI piPSCs

PEFs derived from porcine embryos at day 40 [41] were resuscitated and infected with retrovirus PMX-pOKSM and PMX-IRF1, and control cells were infected with retrovirus PMX-pOKSM and PMX-tdTomato for reprogramming. The piPSCs were induced as described in the previous section. Staining for AP was performed at day 20 of reprogramming. Numbers of AP-positive colonies were statistically analyzed for comparison. PEFs infected with retrovirus at day 3 were also collected for RNA extraction. Cell colonies induced by PMX-POKSM and PMX-IRF1were selected, detached, and seeded on feeder cells to generate more than 3 cell lines, which were named OKSMI piPSCs. Then, the pluripotency of OKSMI piPSCs was detected by in vitro differentiation, immunofluorescence, AP staining, and qRT-PCR.

### AP staining and karyotype analysis

#### AP staining

piPSCs were fixed in 4% paraformaldehyde (3053589-4, Sangon Biotech) at room temperature for 3–5 min and then washed with Dulbecco’s phosphate-buffered saline (DPBS). Fixed cells were incubated in AP staining solution as previously described [41].

#### Karyotype analysis

piPSCs was accomplished by incubation in medium containing KaryoMAX Colcemid Solution (15210-040, Gibco) for 3 h and then digested into single cell suspensions. Cell pellets were resuspended in 10 ml 0.075 M KCl and incubated at 37 °C for 20 min. Next, the KCl solution was added to the pre-chilled fixative solution (methanol in acetic acid, 3:1 v/v). After centrifugation at 500×*g*, cell pellets were resuspended in 10 mL pre-chilled fixative solution and incubated on ice for 30 min. Then, centrifugation at 500×*g* was repeated and pellets were resuspended and incubated on ice for 1 h. The cell pellets were then resuspended in 200 μL liquid and dropped onto microscope slides. After drying, microscope slides were stained with the Rapid Giemsa Staining kit (E6073141, BBI Life Science).

### Immunofluorescence

Cells were fixed with 4% paraformaldehyde for 30 min and washed thrice with DPBS by shaking at 70 rpm for 5 min. The cells were then incubated in 0.5% Triton X-100 for 30 min. Next, the cells were washed with DPBS, and subsequently blocked in blocking solution (P0102, Beyotime) for 1 h. Then, cells were stained with the primary antibody overnight. After washing in DPBS, cells were stained for 1 h with the appropriate secondary antibodies conjugated to Alexa Fluor 488 and washed in DPBS. Finally, cellular nuclei were labeled with DAPI (1:5000, 3–5 min). Fluorescence signals were detected using an inverted fluorescence microscope. Primary and secondary antibodies used here are listed in Table S2.

### Embryoid body (EB) formation and in vitro differentiation

piPSCs were cultured in a 6-well plate to 80–90% confluence. The cells were digested into single cell suspensions and then seeded on 6-cm dishes with shaking at 70 rpm. After EBs were formed, they were plated in 24-well plates for differentiation. After 7–10 days, the expression of lineage differentiation genes was detected by Immunofluorescence microscopy.

### RNA extraction, qRT-PCR, and RT-PCR

Cells collected for RNA extraction were lysed in Trizol® Reagent (15596018, Life Technology) and the total RNA of each sample was extracted according to the manufacturer’s instructions. Next, total RNA was reverse transcribed to cDNA by the 5× All-in-one RT MasterMix (G490, abm). qRT-PCR were performed with the Light Cycler® 480 Instrument (Roche) using the 2× RealStar Power SYBR Mixture (A311-05, Genestar) and the primers used are presented in Table S3. RT-PCR were performed using 2× Es Taq MasterMix (CW0690S, CWbio) and primes are presented in Table S3.

### Transcriptome analysis

#### Transcriptome analysis for transcriptome data of pig ICM and TE

The transcriptome of the porcine ICM and trophectoderm (TE) was sequenced by Liu et al. [45]. The sequencing reads were deposited under accession number GSE139512 in the NCBI GEO database and were re-mapped and analyzed as follows: low-quality reads and adaptor sequences were trimmed with Trimmomatic [46]. Clean reads were aligned to the *Sus scrofa* 10.2 genome (from Ensemble) by Hisat2 [47]. Gene counts were calculated by counting the overlap of reads on each gene with HT-seq [48]. Expression levels were normalized as RPKM with the gene annotation files from the Ensemble (release 94) and edge R package in R [49]. Transcription factors were selected from TFDB [50] according to orthologous genes in mice. Differentially expressed genes (DEGs) were identified using the DESeq2 package. Functional enrichment for Gene Ontology (GO) and KEGG were performed using the GOstats package [51]. Network analysis of DEGs was performed using the STRING database [52].

#### RNA-seq and transcriptome analysis for IRF1 overexpression cells

RNA-seq was performed and the data was analyzed by Tang Tang Biomedical Technology Co., Ltd. (Beijing). Raw data was filtered using trim_galore [46] and used to map to the *Sus scrofa*.11.1 genome (from Ensemble) for mapping of reads by HISAT2 [53]. The data was processed with htseq-count (v0.6.0) [49], to tabulate the read counts of each transcript. The gene expression levels (FPKM) of each sample were calculated using stringtie (version 2.0) [54] and the TPM of each sample was calculated using kallisto (version 0.46.0) [55]. Transcript quantification was performed using featureCounts, a part of the subread package (version 1.6.4) [56] and DEGs were performed using DESeq2 [57] The up- and downregulated gene lists were selected for enrichment of GO terms and KEGG pathways using the online KOBAS 3.0 tool [58]. RNA-seq data of IRF1 overexpressing piPSCs was uploaded to the NCBI Gene Expression Omnibus (GSE143484).

### Chromatin immunoprecipitation

Chromatin immunoprecipitation (ChIP) was performed using the SimpleChIP® Plus Enzymatic Chromatin IP Kit (Magnetic Beads) (9005, CST) according to the manufacturer’s instructions. Briefly, 4 × 10^6^ cells were collected in 1 ml DPBS with 5 μl 200× Protease/Phosphatase Inhibitor Cocktail (PIC). Chromatin was cross-linked by adding 27 μl 37% formaldehyde for 12 min, and the reaction was stopped by the addition of glycine solution. Cell pellets were lysed by sequential incubation in buffer A then buffer B. Next, nuclear processing and chromatin digestion were performed by digesting the cellular nuclei with 0.45 μl Micrococcal Nuclease in 100 μL buffer B for 20 min at 37 °C. Digestion was stopped by the addition of 10 μl 0.5 M EDTA. After centrifugation, nuclear pellets were resuspended in ChIP buffer and disrupted by ultrasonication for 3 rounds of 20 s, followed by a 30-s incubation on ice. Then, 100 μl chromatin sample from the lysed product was used to extract DNA as the input sample and 400 μl of chromatin containing approximately 10 μg DNA was immunoprecipitated at 4 °C overnight with 6 μg of anti-FLAG antibody (F1804-1MG, Sigma) with rotation at 60 rpm. Next, ChIP-Grade Protein G Magnetic Beads in CHIP buffer, and the samples were incubated for 4 h at 4 °C. The chromatin immunoprecipitated was then eluted from the magnetic beads in ChIP elution buffer at 65 °C for 2 h, with shaking at 1200 rpm. Fragments of DNA were purified from the elution buffer using DNA purification spin columns. Input and CHIP DNA samples were submitted to the Wuhan IGENEBOOK Biotechnology Co., Ltd. for library preparation and sequencing. The raw data was uploaded to the NCBI Gene Expression Omnibus (GSE143484).

### ChIP-Seq analysis

For the ChIP-Seq data of Flag tagged genes, cleaned reads were aligned to the *Sus scrofa* genome 11.1 (from Ensemble) using bowtie2 (version 2.2.5) [59] with the default parameters. Signal tracks for each sample were generated using MACS2 (version 2.1.2) [60]. The biological replicates were then pooled together for each group, and downstream analyses were performed [61]. The signal intensity for each sample was calculated, which is defined as ± 2 kb around the transcription start site (TSS) [62]. Annotation and visualization of ChIP peak coverage over the chromosomes was conducted using the ChIPseeker package in R (version 1.18.0) [63]. The annotation file required by ChIPseeker was generated using GenomicFeatures (version 1.34.8) [60].

ChIP-Seq data of H3K4me in EPSCs were downloaded from ArrayExpress (E-MTAB-7252) [19]. The methods used for data align, removing duplicate reads, and calling peaks were the same as the previous part. BAM files were converted to bigWig files by deeptools (vesion 3.3.0) [64] and visualized in IGV (vesion 2.6.0) [65].

### Statistical analyses

All of the data were presented as mean ± standard deviation. Statistical analyses consisted of the Student’s *t* test for two groups. Data was considered statistically significant when *P* < 0.05. Comparisons of multiple groups were analyzed using one-way ANOVA with Tukey’s multiple comparison test. There were three biological replicates in the overexpression and knockdown of IRF-1 during reprograming for the analysis of AP-positive colonies, which was accomplished using the Student’s *t* test.

## Results

### IRF-1 is a specific transcription factor in porcine ICM

**Fig. 1:**
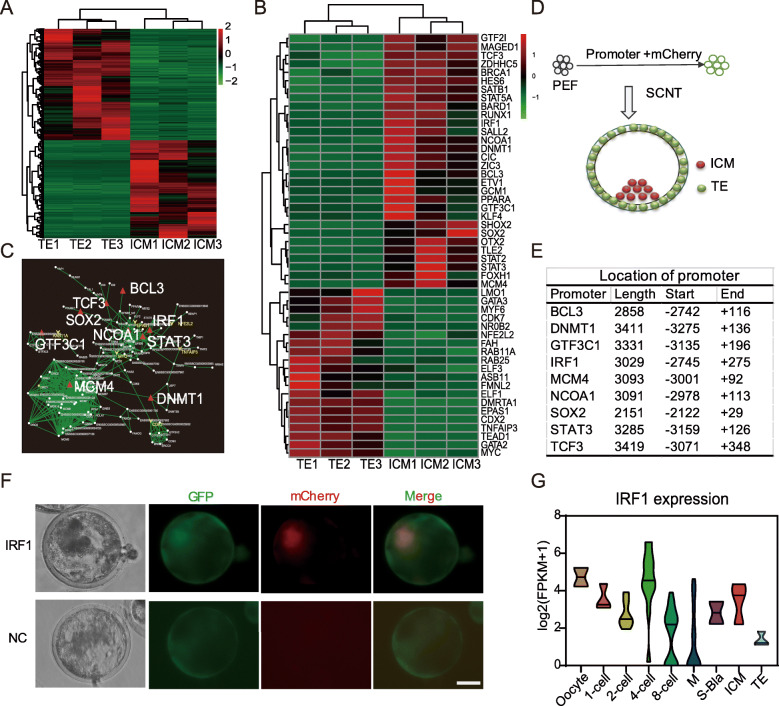
IRF-1 is a specific transcription factor in porcine ICM. **a** Heat map showing differential expression of genes between ICM and TE. **b** Heat map showing differential expression of transcription factors between ICM and TE. **c** Network diagram of transcription factors and DEGs. The red triangles represent upregulated transcription factors in the ICM. The yellow triangles represent upregulated transcription factors in the TE. The white origin represents proteins targeted by the transcription factors. **d** Schematic diagram of construction and verification of reporting system for transcription factors. Reporter cells were established by random insertion of specific promoter driven mCherry cassette and then used as donor cells for SCNT. Red fluorescence in reconstructed blastocysts reflects the location of target gene. **e** Location of the promoter sequences used for reporter construction. **f** Distribution of red fluorescence in porcine IRF-1-mCherry reconstructed blastocysts by SCNT. GFP fluorescence represents successful integration of reporter system. Red fluorescence represents the activity of the promoter activity. Scale bar, 50 μm. **g** Violin plots of IRF1 expression in pig embryos. M, morula; S-Bla, small blastocyst

To achieve characteristic gene expression patterns of the ICM, the transcriptomic data of ICM and TE from Bama miniature pigs [45] were compared. A total of 911 genes were differentially expressed with 426 upregulated in the ICM and 485 upregulated in the TE, respectively (Fig. 1a). Next, KEGG pathways were significantly enriched for the ICM-specific genes, which included signaling pathways regulating pluripotency of stem cells (Figure S1A). Transcription factors of ICM and TE were analyzed, revealing a total of 50 candidate transcription factors, 30 of which were expressed in ICM and 20 in TE (Fig. 1b). The differentially expressed transcription factors upregulated in ICM were associated to the pluripotency and embryonic development, which was proved by GO and KEGG enrichment; thus, 9 transcription factors specifically upregulated in ICM (BCL3, IRF-1, DNMT1, GTF3C1, NCOA1, SOX2, STAT3, TCF3, and MCM4) were selected for further analysis. Protein-protein interaction analyses were performed among the DEGs between ICM and TE and the network implied that key regulatory relationship in ICM (Fig. 1c).

To confirm the expression patterns of these transcription factors in blastocysts, reporter cells were established by random insertion of specific promoters driven mCherry cassette and constitutive GFP expression (Fig. 1d, e). The regions of these genes’ promoters were analyzed using the ChIP-Seq data of H3K4me3 [19], which supported all the region we amplified covered the promoter of respond genes (Fig. 1e, Figure S1B). PEFs transfected with reporter plasmids showed GFP fluorescence as expected (Figure S1C). The PEFs carrying the reporter system were used as donor cells for SCNT. The mCherry fluorescence associated with the expression of IRF-1 was observed in aggregation in blastocysts 6–7 days post-SCNT (Fig. 1d, f). Fluorescence associated with STAT3 and DNMT1 was observed in all blastocysts (Figure S1D). In contrast, fluorescence of all other genes assessed was faint (data not shown).

Furthermore, mCherry fluorescence as activated by BCL3 was punctate and only present in a few blastocyst cells (Figure S1D). However, overexpression of BCL3 in piPSCs that was derived by Zhang et al. [15] showed no significant effect on the expression of pluripotency-associated genes (Figure S1E). As a result, the studies conducted here focused on the potential pluripotency modulating functions of IRF-1. Similarly, transcriptome data from early embryos [45] showed that expression of IRF-1 in vivo exhibits an initial decrease, which increased in the 4-cell embryo and is expressed at higher levels in ICM relative TE (Fig. 1g).

### DOX-hLIF-2i piPSCs were characteristically pluripotent

**Fig. 2:**
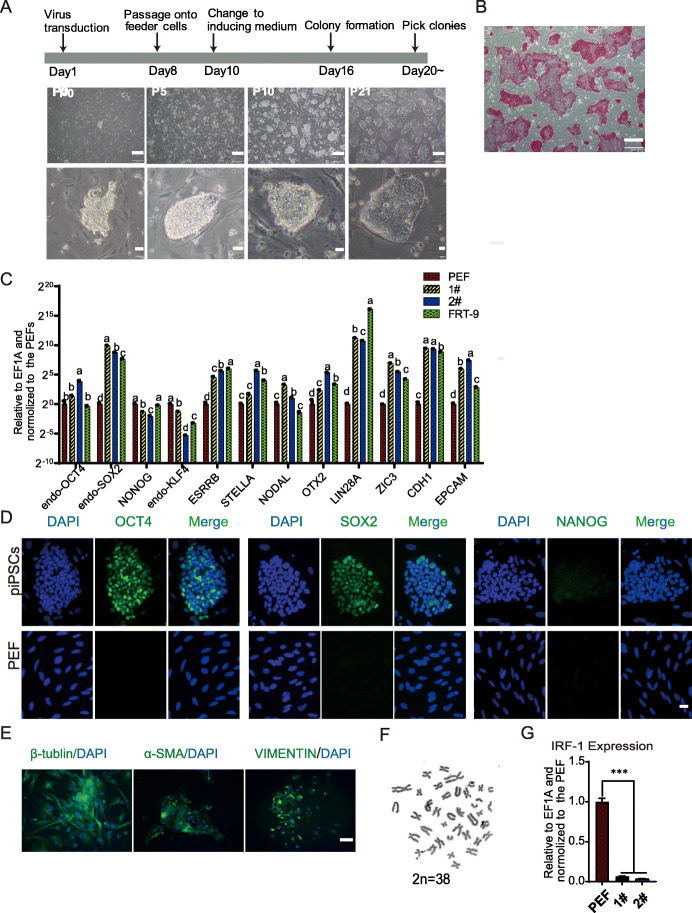
DOX-hLIF-2i piPSCs were characteristically pluripotent. **a** Schematic illustration of DOX-hLIF-2i piPSCs generation and cell morphologies from P0 to P21. **b** AP staining of DOX-hLIF-2i piPSCs. Scale bar, 200 μm. **c** qRT-PCR analysis of pluripotency-associated gene expression. 1# and 2# represent two lines of DOX-hLIF-2i piPSCs. FRT-9 represents naive-like piPSCs derived by Zhang et al. [15]. Comparisons of multiple groups were analyzed using one-way ANOVA with Tukey’s multiple comparison test. Groups with different letters indicate significant differences (*p* < 0.05). **d** Immunofluorescence assay of OCT4, SOX2, and NANOG. Scale bar, 20 μm. **e** Immunofluorescence assay of 3-germ-layer cells in EBs. Scale bar, 100 μm. **f** Karyotype analysis of DOX-hLIF-2i piPSCs. **g** qRT-PCR analysis of IRF-1 in DOX-hLIF-2i piPSCs and PEFs

PEFs containing the OCT4-tdTomato reporter system [44] were used to induce piPSCs, while the inducible lentiviruses FUW-OSKM and Fuw-M2Rtta were used to induce reprogramming. As is presented in Fig. 2a, three homogeneous stable cell lines were established for multiple passages. The resulting cell lines were cultured in hLIF+pLIF+2i+DOX N2/B27 medium, which were named DOX-hLIF-2i piPSCs. The piPSCs were AP-positive (Fig. 2b) and expressed pluripotency genes including SOX2, ESRRB, STELLA, LIN28A, EPCAM, and CDH1 (Fig. 2c). Immunofluorescence also indicated clear OCT4 and SOX2 expression, weak expression of SSEA-4, TRA-1-60, and TRA-1-81, but no expression of NANOG (Fig. 2d, Figure S2A). Then, DOX-hLIF-2i piPSCs were differentiated into EB balls in vitro and these cells expressed 3-germ-layer markers including β-tublin (for ectoderm), α-SMA (for mesoderm), and vimentin (for endoderm) (Fig. 2e and S2B), which indicated DOX-hLIF-2i piPSCs possessed the capacity for differentiation. Furthermore, the piPSCS had normal chromosomal number (2n = 38, XY) (Fig. 2f). Consistent with the flat morphology, DOX-hLIF-2i piPSCs expressed several primed genes including NODAL, OTX2, and ZIC3 (Fig. 2c), as well as the weak primed surface antigens SSEA-4, TRA-1-60, and TRA-1-81. The above results suggest that DOX-hLIF-2i piPSCs were pluripotent as demonstrated.

However, the endogenous OCT4 was expressed at low level, and NANOG was not activated (Fig. 2c, Figure S2E), which was consistent with the results that the fluorescence of OCT4-tdTomato reporter was not observed in DOX-hLIF-2i piPSCs (Figure S2C). RT-PCR showed that although endogenous SOX2 was expressed in DOX-2i-hLIF piPSCs, endogenous KLF4 and cMYC were expressed lower compared with PEFs and the exogenous OSKM was still expressed (Figure S2E). Furthermore, proliferation of the piPSCs ceased and their phenotypes became AP-negative after withdrawing the inducer DOX (Figure S2D). This indicated that self-renewal of DOX-hLIF-2i piPSCs is reliant on the exogenous OSKM. Therefore, the pluripotency state of DOX-hLIF-2i piPSCs can still be further improved. Moreover, the expression of IRF-1 was lower in DOX-hLIF-2i piPSCs than PEFs (Fig. 2g) and the FPKM of IRF-1 in piPSCs was 1.41 ± 0.01 that was included in Table S4, which illustrated the trace expression level of IRF-1.

### Overexpression of IRF-1 promotes pluripotency in DOX-hLIF-2i piPSCs

**Fig. 3:**
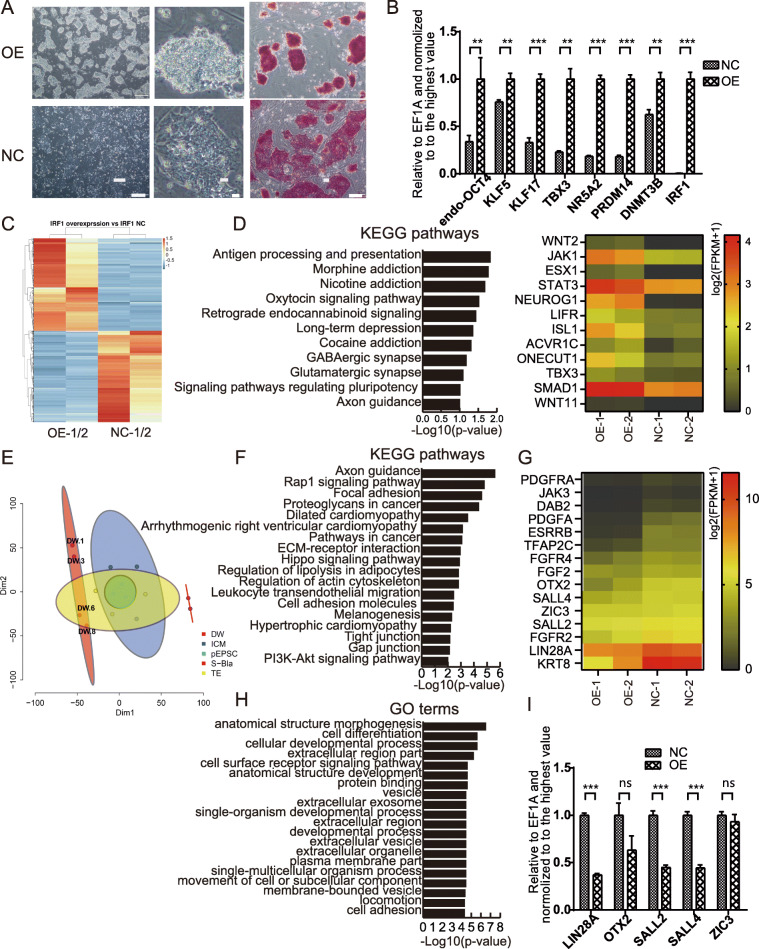
Impact of IRF-1 overexpression on DOX-hLIF-2i piPSCs. **a** Cell morphologies and AP staining of IRF-1 overexpressing and control cells. Scale bars from left to right, 200 μm, 20 μm, and 100 μm. OE, IRF-1 overexpressing cells; NC, negative control. **b** qRT-PCR analysis of pluripotency-associated genes in IRF-1 overexpressing and control cells. **p < 0.01; ***p < 0.001. OE, IRF-1 overexpressing cells; NC, negative control. **c** Heat map showing differential expression of genes between IRF-1 overexpressing and control cells. OE, IRF-1 overexpressing cells; NC, negative control. **d** KEGG pathways enriched from upregulated genes in the IRF-1 overexpressing cells. The heat map shows genes enriched for signaling pathways regulating pluripotency in IRF-1 overexpressing cells. **e** The t-SNE map of IRF-1 overexpressing cells and control cells, the ICM and TE of pig blastocysts, and EPS cells. The transcriptomic profiles of ICM, TE of pig embryos are from the dataset published by Liu et al. [45]. The transcriptomic profiles of EPS cells are from the dataset published by Liu et al. [19]. DW-1/3, IRF-1 overexpression cells; DW-6/8, control cells. **f** KEGG pathways enriched from downregulated genes in the IRF-1 overexpressing cells. **g** Heat map showing genes downregulated in the IRF-1 overexpressing cells. **h** GO terms enriched from downregulated genes in the IRF1 overexpressing cells. **i** qRT-PCR analysis of primed genes in IRF-1 overexpressing and control cells. **p < 0.01; ***p < 0.001; ns, no significance

Given that IRF-1 is expressed only in trace amounts in DOX-hLIF-2i piPSCs, the gene was overexpressed in these cells to more clearly investigate the effects of IRF-1 on pluripotency of piPSCs. Notably, overexpression of IRF-1 transformed piPSCs from a loose to a tight colony phenotype and enhanced AP staining (Fig. 3a). Moreover, DAPI staining also showed that cell nuclei became smaller and the cells aggregated in the IRF-1 overexpression group (Figure S3A). Analysis of gene expression by qRT-PCR showed that naïve genes OCT4, KLF5, KLF17, TBX3, NR5A2, PRDM14, and DNMT3B were all upregulated (Fig. 3b). RT-PCR showed that endogenous expression of OCT4 was significantly upregulated in the IRF-1 overexpressing piPSCs, while endogenous expression of SOX2, KLF4, cMYC, and exogenous OKSM have no obvious change (Figure S3B). Thus, it appears as though overexpression of IRF-1 promotes the pluripotency of DOX-hLIF-2i piPSCs.

To further investigate the impact of IRF-1 overexpression on pluripotency of piPSCs, the transcription profiles of IRF-1 overexpressing and control cells were analyzed. Approximately 1200 genes were upregulated, and 1424 genes were downregulated in the IRF-1 overexpressing DOX-hLIF-2i piPSCs (Fig. 3c, Table S4). Among the upregulated genes, the enriched KEGG pathways included signaling pathways regulating pluripotency of stem cells, which supports what is known for the roles of WNT2, JAK1, STAT3, LIFR, TBX3, and SMAD1 (Fig. 3d). These observations are consistent with the pluripotency promoting effect of IRF-1 overexpression.

Furthermore, these downregulated genes were enriched individually for the relevant GO terms and KEGG pathways. The KEGG pathways were specific to axon guidance, rap1 signaling pathway, dilated cardiomyopathy pathway, and the Hippo signaling pathway (Fig. 3f). All of these pathways are believed to contribute to the promotion of cellular differentiation. In addition, GO terms such as anatomical structure morphogenesis, cell differentiation, cellular developmental process, anatomical structure development, single-organism developmental process, developmental process, and single-multicellular organism process were enriched from downregulated genes (Fig. 3h). Likewise, the transcriptomic profiles of IRF-1 overexpressing cells were compared with that of ICM as well as TE of pig embryos from the dataset published by Liu et al. [45], and porcine EPS cells [19]. The t-SNE map suggested that IRF-1 overexpressing cells deviate from TE cells, while the control cells were in the region of TE cells (Fig. 3e). Similarly, KRT8, DAB2, and TFAP2C regulating the self-renew of trophoblast stem (TS) cells were downregulated (Fig. 3g). Additionally, LIN28A, OTX2, SALL2, FGF2, and FGFR4, which have been associated with primed states, as well as PDGFA and PDGFRA that regulate primitive endoderm differentiation, were downregulated (Fig. 3g, i). These observations suggest that IRF-1 overexpression antagonizes the differentiation of piPSCs.

### High-level expression of IRF-1 was associated with higher expression of naïve pluripotency-associated genes in DOX-hLIF-2i piPSCs

**Fig. 4:**
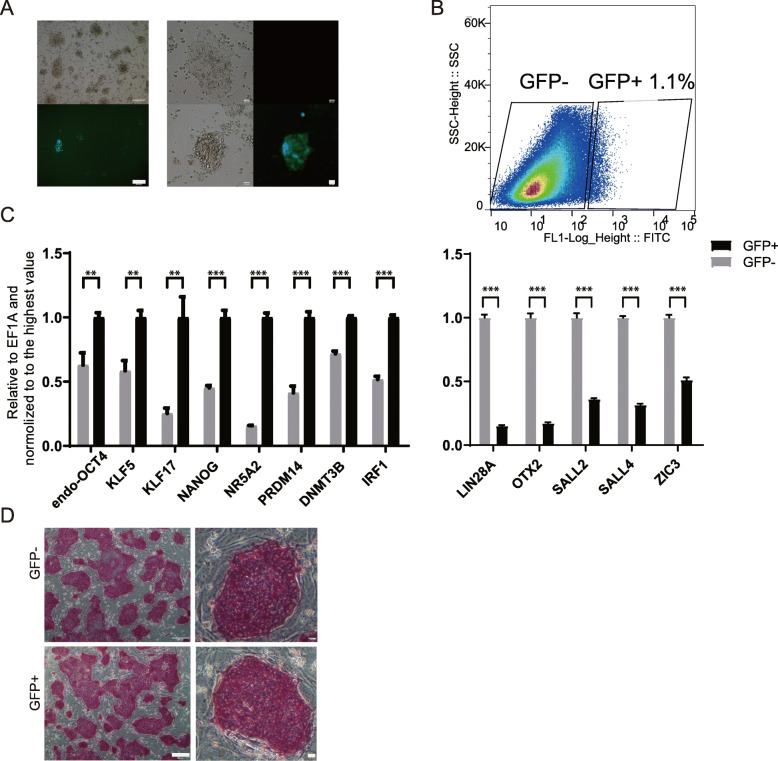
High-level expression of IRF-1was associated with higher expression of naïve pluripotency-associated genes in DOX-hLIF-2i piPSCs. **a** detection of IRF-1-GFP in DOX-hLIF-2i piPSCs. Scale bars from left to right, 200 μm, 20 μm. **b** FACS analysis of GFP-positive cells in DOX-hLIF-2i piPSCs. **c** qRT-PCR analysis of genes in the GFP-positive and -negative cells. **p < 0.01; ***p < 0.001. **d** AP staining of GFP-positive and -negative cells. GFP- represents GFP negative cells and GFP+ represents GFP positive cells

Although the expression of IRF-1 was faint in DOX-hLIF-2i piPSCs (Fig. 2g), it was hypothesized that IRF-1 is expressed in a heterogeneous manner in piPSCs. Therefore, DOX-hLIF-2i piPSCs cells were transfected with PT2-Neo-IRF-1-GFP and pCMV (CAT) T7-SB100 to label IRF-1 with GFP. After selection of stably transfected cells with G418, sporadic GFP fluorescence was observed in DOX-hLIF-2i piPSCs (Fig. 4a). Next, GFP-positive and -negative cells were sorted for qRT-PCR analysis (Fig. 4b, c). Based on this assay, the data provided corroborating evidence that IRF-1 was expressed at higher levels in the GFP-positive cells. Expression of naïve genes such as endogenous OCT4, KLF5, KLF17, NR5A2, PRDM14, and DNMT3B was higher in the GFP-positive cells, while the expression of primed genes, such as LIN28A, OTX2, SALL2, and ZIC3 was lower. Intriguingly, the same variation of pluripotency-associated genes resulting from overexpression of IRF-1 was observed in DOX-hLIF-2i piPSCs (Fig. 3b, i). However, AP staining of the two cell types was indistinguishable (Fig. 4d). After multiple passages, some GFP negative cells appeared among GFP positive population, while some GFP negative population showed a few GFP positive cells (Figure S4), which illustrated that the expression of IRF-1 is transient in these piPSCs. Consequently, it appears as though IRF-1 is associated with porcine naïve pluripotency in DOX-hLIF-2i piPSCs.

### IRF-1 binds genes related to JAK-STAT signaling pathway in DOX-hLIF-2i piPSCs

**Fig. 5:**
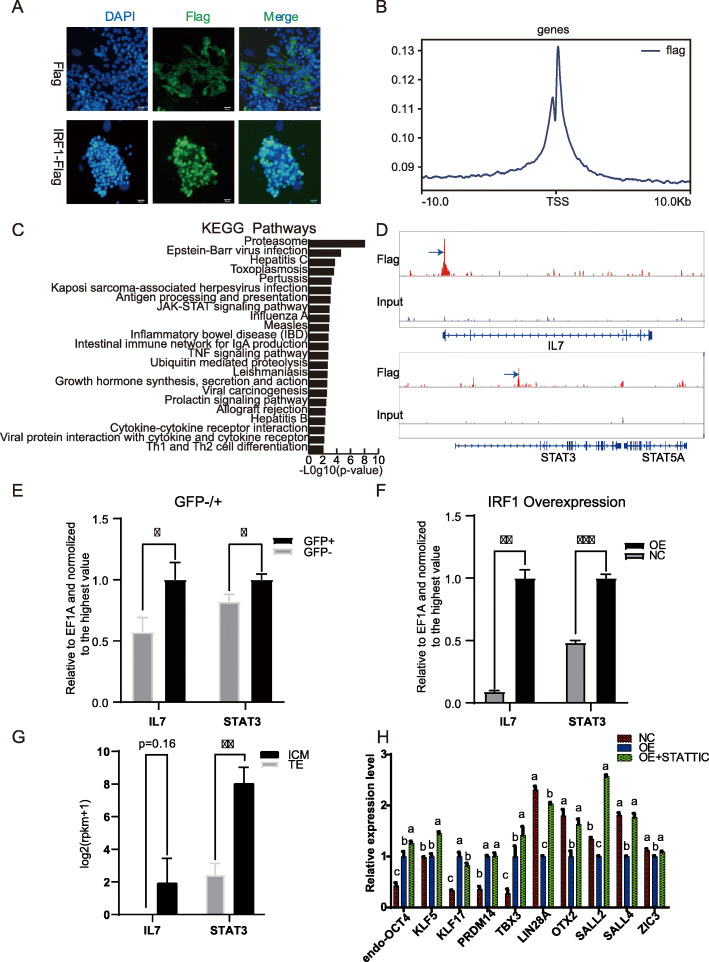
ChIP-Seq analysis for genes targeted by IRF-1. **a** Immunofluorescence co-localization of IRF-1 with FLAG antibody. IRF-1 was overexpressed fusion with 3× Flag. 3× Flag was alone expressed as a control. Scale bar, 20 μm. **b** The profiles of peak signals of IRF-1-Flag. Signals are shown for 10 kb up- and downstream of the TSS. **c** KEGG pathways enriched from IRF-1 targeted sites. **d** Peaks related to IL7, STAT3. Flag denotes signals detected with the anti-flag antibody in IRF-1-Flag overexpressing cells. **e** qRT-PCR analysis of genes in the GFP-positive and -negative cells. *p < 0.05; **p < 0.01; ***p < 0.001. **f** qRT-PCR analysis of genes in the IRF-1 overexpressing cells and control cells. *p < 0.05; **p < 0.01; ***p < 0.001. **f** The expression of genes in the ICM and TE. **p < 0.01. The rpkm was analyzed from transcriptome data sequenced by Liu group. **h** qRT-PCR of genes in IRF-1-overexpressing cells treated with Stattic. NC represents control piPSCs. OE represents IRF-1-overexpressing cells. OE+Stattic represents IRF-1-overexpressing cells treated with stattic for 3 days. Comparisons of multiple groups were analyzed using one-way ANOVA with Tukey’s multiple comparison test. Groups with different letters indicate significant differences (*p* < 0.05)

The subcellular localization of IRF-1 was observed to be in the nucleus in DOX-hLIF-2i (Fig. 5a). This observation was expected, as it functions as a transcription factor. In order to detect the target genes of IRF-1, ChIP-Seq was conducted using an anti-flag antibody and the chromatin of IRF-1-flag overexpressing cells. As can be seen in Fig. 5b, peaks Flag captured were associated with transcription start sites (TSSs). A total of 348 genes within 10 kb of transcriptional start sites were detected. These peak regions and their proximity to NCBI-designated genes are compiled in Table S5. Next, the captured genes were used to enrich for KEGG pathways (Fig. 5c). The most enriched biological pathways were related to initiate immunity and adaptive immunity. Interestingly, the JAK-STAT signaling pathway and Cytokine-cytokine receptor interaction pathways were enriched. Among these pathways, STAT3 and IL7 were included (Fig. 5d). Expression of these genes was also upregulated in the IRF-1 overexpressing cells and IRF-1-GFP-positive cells (Fig. 5e, f). These data suggest that IRF-1 binds these genes, in turn activating their expression.

In addition, STAT3 and IL7 were expressed in porcine ICM, which is based on the transcriptomic data obtained from porcine ICM and TE (Fig. 5g). These observations are consistent with the expression of IRF-1 in ICM. In order to confirm the effect of IL7 on the pluripotency of piPSCs, recombinant IL7 protein was added to hLIF+pLIF+2i+DOX N2/B27 medium at 0 ng/μl, 10 ng/μl, and 25 ng/μl. No obvious changes in the cell morphology and AP staining results were observed, and expression of pluripotency related genes did not significantly change (Figure S5A, B). Therefore, it was inferred that IL7 has no effect on the pluripotency of piPSCs, which also excluded the effect of IL7 on mediating the role of IRF-1’s promotion of pluripotency.

To confirm the roles of the JAK-STAT3 signaling pathways in mediating the regulation of pluripotency by IRF-1’s overexpression, DOX-hLIF-2i piPSCs overexpressing IRF-1 were treated with the STAT3 phosphorylation inhibitor, Stattic. Although there was no significant change in the cell morphology and AP staining which was not shown, the primed genes such as LIN28A, OTX2, SALL2, and SALL4 were upregulated (Fig. 5h). This indicated that IRF-1 downregulated the primed genes by enhancing the phosphorylation of STAT3.

### IRF-1 is a positive regulator during reprogramming

**Fig. 6:**
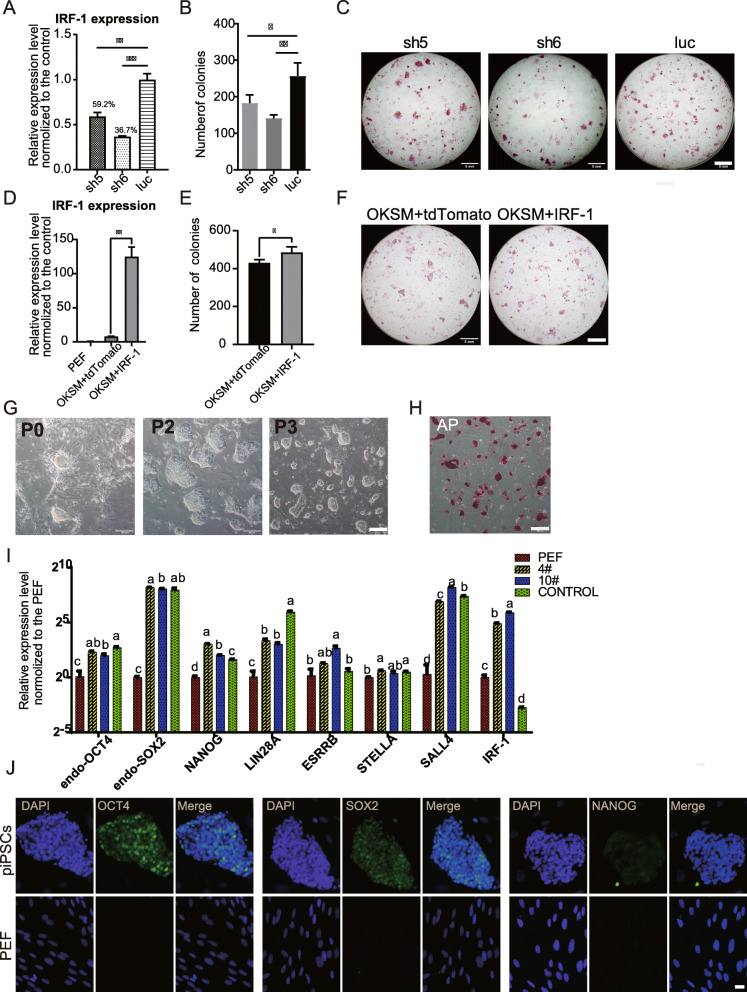
IRF-1 is a positive regulator for reprogramming. **a**–**c** The impact of IRF-1 knockdown on reprogramming. **d**–**f** Impact of IRF-1 overexpression during reprogramming. **g**–**j** The detection of pluripotency in PMX-POKSMI piPSCs. **a**, **d** qRT-PCR analysis of IRF-1 expression at day 5 of reprogramming. **b**, **e** Number of AP+ colonies at day 20 of reprogramming. The numbers are presented as mean values ± SD from 3 independent experiments. **c**, **f** AP+ colonies of pre-piPSCs at day 20 of reprogramming. Scale bar, 5 mm. **g** Morphologies of OKSMI piPSCs from P0 to P3. Scale bar, 200 μm. **h** AP staining of OKSMI piPSCs. Scale bar, 200 μm. **i** qRT-PCR analysis of pluripotency-associated genes of OKSMI piPSCs. 4# and 10# represent different lines of OKSMI piPSCs. The control represents OKSM piPSCs. Comparisons of multiple groups were analyzed using one-way analysis ANOVA with Tukey’s multiple comparison test. Groups with different letters indicate significant differences (*p* < 0.05). **j** Immunofluorescence assay of OCT4, SOX2, NANOG. Scale bar, 20 μm

For the enhancement of pluripotency in the IRF-1 overexpressing piPSCs, the hypothesis was proposed that IRF-1 has an effect on the reprogramming process. Viruses packaged from plasmid PLVTH-shRNA were used along with the viruses (PMX-OSKM) to transduce the cells for knockdown of IRF-1 during reprogramming. Reduced expression of IRF-1 relative to control cells confirmed the efficacy of the shRNAs (Fig. 6a). The number of AP-positive colonies decreased significantly after IRF-1 knockdown (Fig. 6b, c), indicating that IRF-1 is important for reprogramming in porcine stem cells.

Next, the influence of IRF-1 overexpression on reprogramming was tested. To accomplish this, PEFs were co-transduced with PMX-IRF-1 and PMX-pOKSM viruses for reprogramming. Compared with the control, there was a slight improvement observed in the number of colonies overexpressing IRF-1 (Fig. 6d–f). This indicates that overexpression of IRF-1 exerts positive effects on reprogramming. Furthermore, the piPSCs induced by PMX-OSKM and PMX-IRF-1 retroviruses exhibited the clone-like morphology like pluripotent stem cells (Fig. 5g). As expected, the OSKMI piPSCs were AP-positive (Fig. 6h) and expressed the pluripotency-associated genes endogenous OCT4, endogenous SOX2, NANOG, LIN28A, ESRRB, and SALL4 (Fig. 6i, Figure S6A). RT-PCR showed endogenous expression pattern of KLF4, cMYC in OSKMI piPSCs was same as in DOX-hLIF-2i piPSCs and the exogenous expression of OSKM was still not silenced (Figure S6A). Furthermore, immunofluorescence assays showed considerable expression of OCT4, SOX2, and SSEA-4, and weak expression of NANOG, TRA-1-60, and TRA-1-81 (Fig. 6j and Figure S6B). Embryoid body formation and in vitro differentiation indicated that OSKMI piPSCs could give rise to endoderm-, mesoderm-, and ectoderm-like cells (Figure S6C). Taken together, these results suggest that IRF-1 is a positive regulator during reprogramming.

## Discussion

Naïve porcine ESCs or iPSCs, which could be used to produce germline chimeric fetuses, have yet to be established. Although there have been many reports about the derivation of piPSCs, these cells lacked naïve essential features, such as expression of TBX3 and KLF4, and the self-renewal of some cell lines were dependent on exogenous OKS [19, 41, 66, 67]. In the present study, DOX-hLIF-2i piPSCs derived using naïve culture medium 2i+hLIF were AP-positive; expressed SOX2, ESRRB, STELLA, and CDH1; and were capable of differentiation in vitro. However, these cells lacked expression of NANOG and KLF4, no OCT4-tdtomato-positive cells were observed, and they differentiated upon withdraw of the inducer DOX, which suggested the self-renewal of these piPSCs could not be maintained without exogenous OKSM. For the establishment of authentic naïve piPSCs, the transcriptional regulatory network of porcine pluripotent stem cells requires exploration. It has been reported that ICM or EPI cells at the early blastocyst stage in mice, humans, and nonhuman primates exhibit transcriptomic features of naïve pluripotency [20, 26–28]. Based on these reports, transcription factors (TFs) expressed higher in the ICM than TE of porcine blastocysts were analyzed, and IRF-1 was demonstrated to enhance the naïve pluripotency of piPSCs. This observation supports the established view that naïve pluripotent stem cells are direct counterparts of early embryonic cells and the core transcription factors and signaling pathways that regulate porcine naïve pluripotent stem cells might be based on the initial state of ICM in vivo.

In the present study, overexpression of IRF-1 promoted the morphology, AP staining, and expression profiles of pluripotency-associated genes as would be expected when cells approach the naïve state. In wild-type piPSCs, high-level fluorescence activated by the IRF-1 promoter was associated with higher expression of naïve pluripotency related genes. These results suggest a correlation between IRF-1 and porcine naïve pluripotency in piPSCs. Furthermore, transcriptomic analysis indicated that overexpression of IRF-1 attenuates the expression of genes known to promote the process of differentiation. Among these downregulated genes, LIN28A, OTX2, SALL2, FGF2, and its receptor FGFR4 have been demonstrated to play central roles in the self-renewal of primed human and mouse pluripotent cells [68–71]. Additionally, KRT8, DAB2, and TFAP2C are reportedly expressed in TS cells [72, 73], as well as PDGFA and PDGFRA, which are believed to regulate the differentiation from ICM to primitive endoderm cells [74, 75]. These findings would suggest that IRF-1 overexpression antagonizes the differentiation of piPSCs. In summary, we conclude that IRF-1 expressed in the ICM of porcine early blastocyst enhances the pluripotency of piPSCs.

It is well established that IRF-1 is involved in the response to viral infections. Specifically, activation of IRF-1 leads to the activation of IFN-β, thereby initiating a rapid proinflammatory cytokine response [32, 76]. In the present study, JAK-STAT and cytokine-cytokine receptor interaction pathways were enriched from the target sites bound by IRF-1. The JAK-STAT pathway is a multi-ligand and receptor-binding signaling pathway that can be activated by many inflammatory cytokines, such as IL-6 and LIF [77]. It was reported that the induction of the JAK-STAT pathway is considered to be essential for maintaining the naive state of human and mouse pluripotent cells [68, 78]. Moreover, transcriptome data from porcine early embryos shows that the JAK-STAT pathway plays an important role in maintaining the naive state [30]. In our study, among genes targeted by IRF-1, IL7 and STAT3 were demonstrated to be bound and activated by IRF-1 in the IRF-1 overexpressing piPSCs. It was previously determined that IL7 binds the receptor, which in turn activates STAT3 [77]. However, the data presented here failed to corroborate the promotion of pluripotency in piPSCs by IL7. This is likely due to low expression level of the IL7 receptor in DOX-hLIF-2i piPSCs. In the present study, an inhibitor of STAT3 phosphorylation reverted the expression of primed genes in IRF-1 overexpressing cells to the higher levels observed in the WT cells. This indicates that IRF-1 inhibited the primed state by binding and activating those involved in the JAK-STAT pathway. However, the mechanism of IRF-1 promotion of the naïve state in piPSCs requires further examination.

In this study, knockdown of IRF-1 during reprogramming reduced reprogramming efficiency. In contrast, overexpression improved the reprogramming efficiency. These results indicated that IRF-1 is a positive regulator of programming. Furthermore, we demonstrated that IRF-1 activates the JAK-STAT signaling pathway and promotes pluripotency of piPSCs. Activation of STAT3 has been reported and could significantly promote the reprogramming from MEFs to iPSCs and have an effect on DNA demethylation of pluripotent loci including Oct4, Nanog, and the imprinting of Dlk1-Dio3 regions and open-chromatin formation during late-stage reprogramming [79, 80]. Therefore, it is speculated that activation of IRF-1 might enhance reprogramming through promoting the activation of the JAK-STAT pathway. In agreement with this proposed mechanism, IRF-1 has been reported to express from the middle to the late stage of reprogramming [38]. Furthermore, it has also been reported that activation of innate immunity is required for efficient nuclear reprogramming. As such stimulation of TLR3 causes rapid and global changes in the expression of epigenetic modifiers to enhance chromatin remodeling and nuclear reprogramming [10]. In addition, IRF-1 was reported to play important roles on the immune response [13–15]. So it is speculated that in addition to promoting reprogramming by JAK-STAT, IRF-1 might promote global changes in the expression of epigenetic modifiers by initiating immune responses, thereby enhancing reprogramming. However, this hypothesis requires experimental verification. Therefore, it is possible that virus infection could induce the expression of IRF-1 [81–84]. The enhancement of reprogramming by virus infection might be partially due to the activation of JAK-STAT downstream of IRF-1 during reprogramming, which might enrich the mechanism of enhancement by retrovirus mediated gene overexpression during reprogramming [85]. Instead of retroviral overexpression, treatment with cytokines for activation of IRF-1 during reprogramming might be an appropriate method for enhancing reprogramming for more safe transgene-free iPSCs.

Porcine naïve stem cells with germline chimerism have yet to be successfully established. The reporting system used to study the naïve state and indicate the pluripotent state has received little attention, with the exception of the OCT4-tdTomato reporter [44]. It was demonstrated here that the fluorescence of mCherry under the control of the IRF-1promoter was observed in aggregation of 6~7 days SCNT blastocysts. To obtain additional information on the ability of IRF-1 to enhance the pluripotency in piPSCs, fluorescent genes activated by the IRF-1 promoter might be a good system for probing the pluripotent state of porcine stem cells.

## Conclusions

Highly interconnected TFs of porcine early ICM were analyzed from the transcriptome data of porcine ICM and IRF-1 was screened for ICM-specific expression, which might regulate the pluripotency of piPSCs. Overexpression of IRF-1 in piPSCs resulted in increased naïve pluripotency as well as an inhibition of genes and pathways related to differentiation. Moreover, the heterogeneity of IRF-1 was observed to be associated with naïve pluripotency in DOX-hLIF-2i piPSCs. ChIP-seq suggested that IRF-1 could bind genes associated with the JAK-STAT signaling pathway, which was consistent with that the inhibitor of STAT3 phosphorylation reverted the expression of primed genes in IRF-1 overexpressing cells. In addition, knockdown and overexpression of IRF-1 during reprogramming suggested that IRF-1 is a positive regulator of reprogramming. In summary, our findings illustrate the ability of IRF-1 to enhance the pluripotency of piPSCs, in part through promoting the JAK-STAT pathway.

## Supplementary Information

**Additional file 1: Figure S1.** Screening for potential transcription factors related to porcine pluripotency, related to Fig. 1. **(A)** GO terms and KEGG pathways enriched from upregulated genes in ICM.**(B)** Analysis of ChIP-Seq data of H3K4me3. Marks indicate promoter regions of selected transcript factors. ChIP-Seq data of H3K4me3 in pEPSCs was obtained by Liu et al. [19]. The peaks from -4 k to -5 k upstream from the transcriptional start site of IRF-1 were peaks associated with other genes not IRF-1. **(C)** GFP fluorescence in PEFs transfected with reporter plasmid. Scale bar, 500 μm.**(D)** Distribution of red fluorescence in porcine reconstructed blastocysts by SCNT. GFP fluorescence represents successful integration of the reporter system. Red fluorescence represents the promoter’s activity. Scale bar, 100 μm.**(E)** qRT-PCR analysis of pluripotency-associated genes in BCL3 overexpressing cells and controls. **Figure S2.** Pluripotency characterization of DOX-hLIF-2i piPSCs, related to Fig. 2. **(A)** Immunofluorescence assay of SSEA-4, TRA-1-60, TRA-1-80. Scale bar, 20 μm. **(B)** EBs of DOX-hLIF-2i piPSCs obtained at day 6 of differentiation. Scale bar, 100 μm. **(C)** Fluorescence detection of OCT4-tdTomato in DOX-hLIF-2i piPSCs. Scale bar of the top figure, 100 μm. Scale bar of the bottom figure, 50 μm. (**D)** Cell morphology and AP staining of DOX-hLIF-2i piPSCs with DOX and without DOX. Scale bar, 200 μm. **(E)** RT-PCR analysis of endogenous expression of OCT4, SOX2, KLF4 and cMYC and exogenous OKSM. EF1A was used as internal control. 1#, 2# represent two lines of DOX-hLIF-2i piPSCs. **Figure S3.** The effect of IRF-1 overexpression on DOX-hLIF-2i piPSCS morphology, related to Fig. 3. **(A)** DAPI staining of IRF-1-overexpressing and negative control piPSCs in Fig. 3a. Scale bars from left to right, 200 μm, 50 μm. **(B)** RT-PCR analysis of endogenous expression of OCT4, SOX2, KLF4 and cMYC and exogenous OKSM. EF1A was used as internal control. OE: IRF-1 overexpressing piPSCs, WT: DOX-hLIF-2i piPSCs. **Figure S4**. Detection of heterogeneity stability of IRF-1 in DOX-hLIF-2i piPSCs, related to Fig. 4. **(A)** Fluorescence detection of GFP positive and negative cells after passage. Scale bars from left to the right, 100 μm, 200 μm. **Figure S5.** The effect of treatment with IL7 or Stattic treatment on pluripotency of DOX-hLIF-2i piPSCs, related to Fig. 5. **(A)** Cell morphology and AP staining of DOX-hLIF-2i piPSCs after treatment with IL7. Scale bars, 200 μm. **(B)** qRT-PCR analysis of pluripotency associated genes in piPSCS treated with IL7. *, *P* < 0.05; **, *p* < 0.01; ***, *p* < 0.001. **Figure S6**. Further Characterization of OSKMI piPSCs, related to Fig. 6. **(A)** RT-PCR analysis of endogenous expression of OCT4, SOX2, KLF4 and cMYC and exogenous OKSM. EF1A was used as internal control. 4#, 10# represent different lines of OKSMI piPSCs. The control group represents OKSM piPSCs. **(B)** Immunofluorescence of SSEA-4, TRA-1-60, TRA-1-80 in OSKMI piPSCs. Scale bar, 20 μm.**(C)** Immunofluorescence assay of 3-germ-layer cells in EBs. Scale bar, 50 μm.

**Additional file 2: Table S1.** Primers used for vector construction.

**Additional file 3: Table S2.** Primary antibody used for Immunofluorescence.

**Additional file 4: Table S3.** Primers for qRT-PCR and RT-PCR.

**Additional file 5: Table S4.** Differentially expressed genes between IRF-1 overexpression and control cells.

**Additional file 6: Table S5.** Target sites and genes associated with peaks from ChIP-Seq.

## Data Availability

All data generated or analyzed in this study are included in this published article. Data of RNA-seq and ChIP-seq in this study were submitted to the NCBI Gene Expression Omnibus under accession number GSE143484.

## Abbreviations

AP: Alkaline phosphatase
CHIP: Chromatin immunoprecipitation
ChIP-Seq: Chromatin immunoprecipitation followed by deep sequencing
CHO: Chinese hamster ovary
COCs: Cumulus oocyte complexes
DMEM: Dulbecco’s modified Eagle medium
DEGs: Differentially expressed genes
DOX-hLIF-2i piPSCs: Porcine-induced pluripotent stem cells induced by doxycycline-inducible expression of defined factors and cultured in medium containing PD0325901, CHIR99021, and human LIF
DPBS: Dulbecco’s phosphate-buffered saline
EB: Embryoid body
EPI: Epiblasts
ESCs: Embryonic stem cells
FPKM: Fragments per kilobase of exon model per million mapped fragments
GFP: Green fluorescence protein
GO: Gene Ontology
ICM: Inner cell mass
IRF-1: Interferon regulatory factor 1
JAK-STAT: Janus kinase/signal transducers and activators of transcription
KCl: Potassium chloride
KEGG: Kyoto Encyclopedia of Genes and Genomes
KOSR: Knockout serum replacement
LIF: Leukemia inhibitory factor
OKSMI piPSCs: piPSCs induced by OCT4, KLF4, SOX2, MYC, and IRF-1
PEFs: Porcine embryonic fibroblasts
PIC: Protease/phosphatase inhibitor cocktail
piPSCs: Porcine-induced pluripotent stem cells
qRT-PCR: Quantitative real-time PCR
RPKM: Reads per kilobase of exon model per million mapped reads
SCNT: Somatic cell nuclear transfer
TE: Trophectoderm
TFs: Transcription factors
TPM: Transcripts per kilobase of exon model per million mapped reads
TS: Trophoblast stem
TSSs: Transcription start sites
t-SNE: t-Distributed stochastic neighbor embedding
