# Supplementary figures and images for: IRF-1 expressed in the inner cell mass of the porcine early blastocyst enhances the pluripotency of induced pluripotent stem cells
Source: Stem Cell Res Ther. 2020 Nov 27;11:505. doi: 10.1186/s13287-020-01983-2 (PMC7694439; doi:10.1186/s13287-020-01983-2)

Figure S1

A

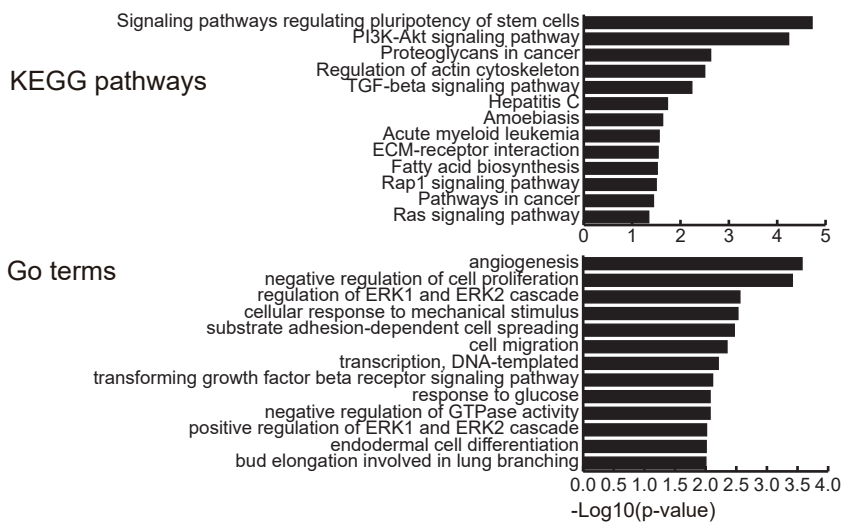

B

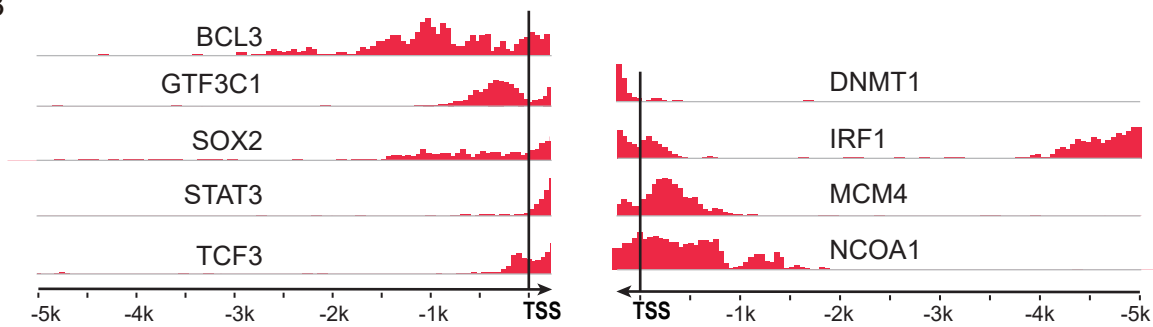

C

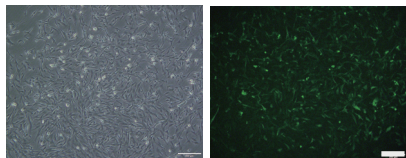

D

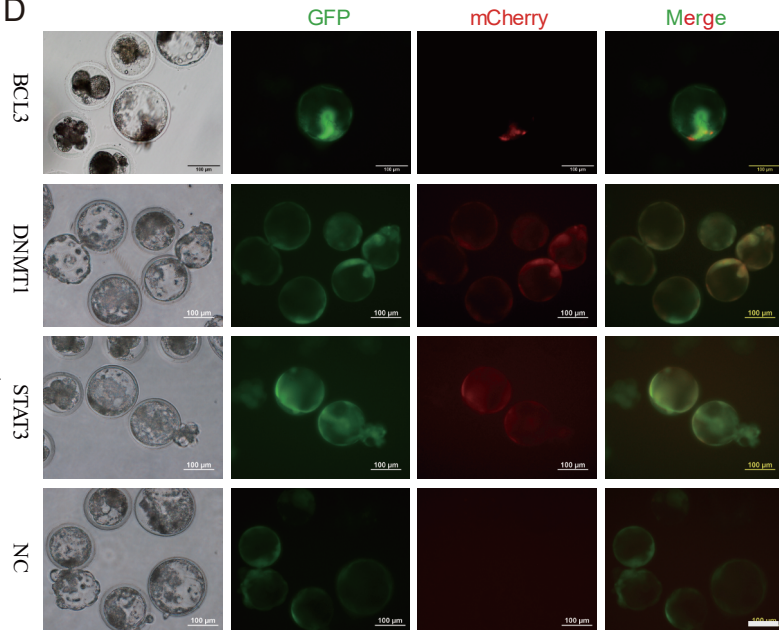

E

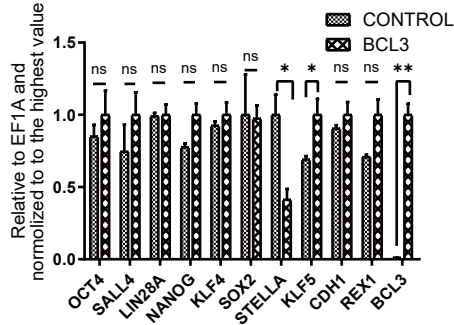

A

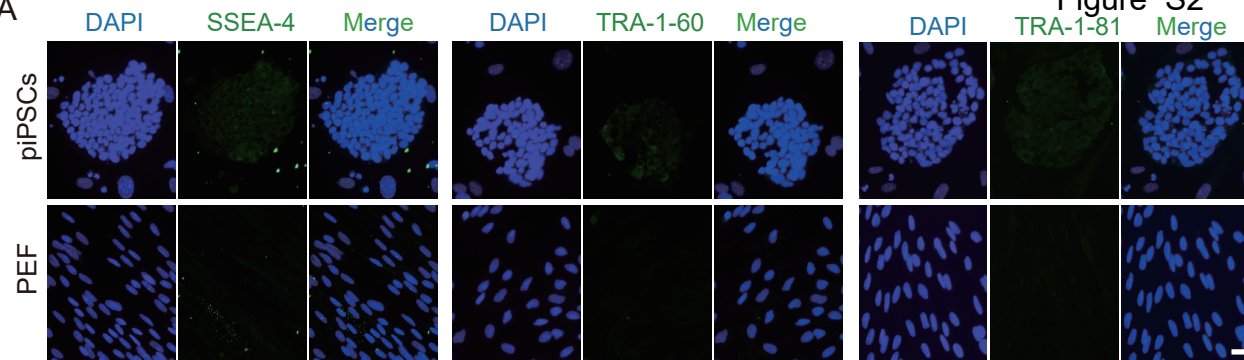

B

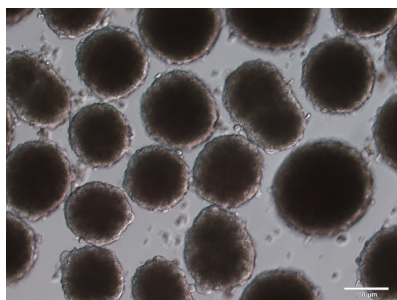

C

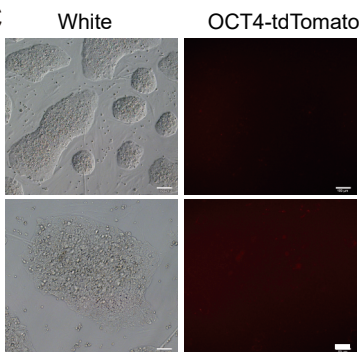

D

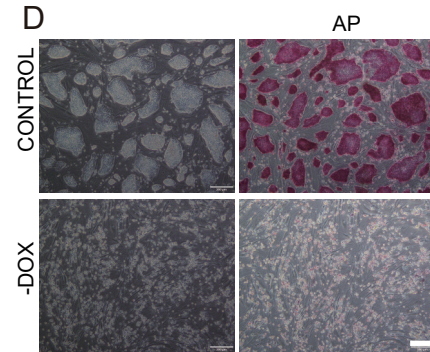

E

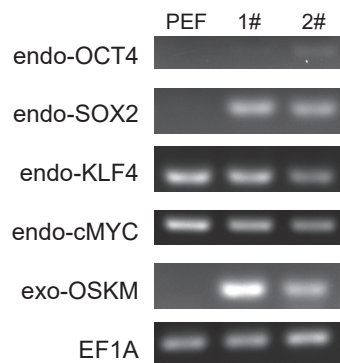

A

OE

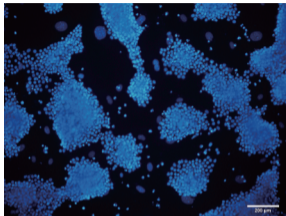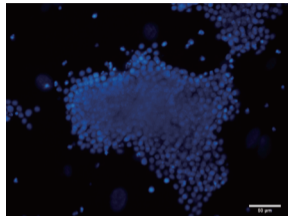

NC

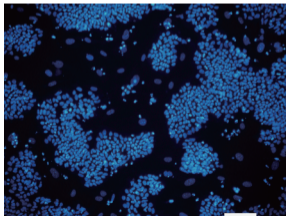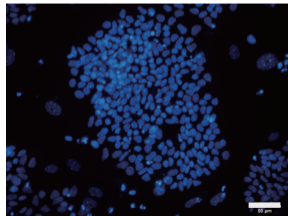

B

Figure S3

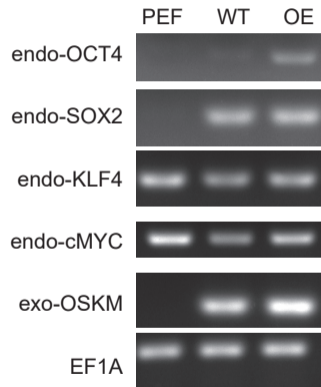

A

Sort-P2

GFP-

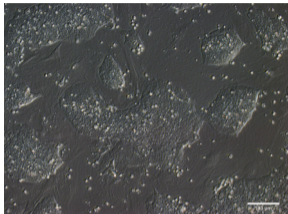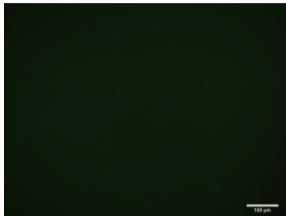

GFP+

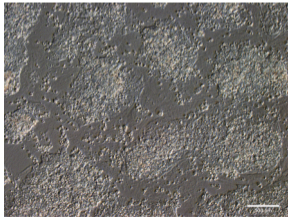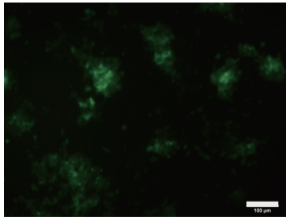

Sort-P3

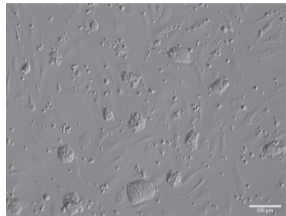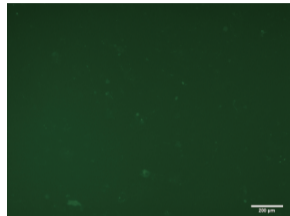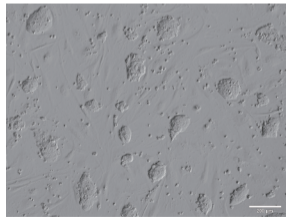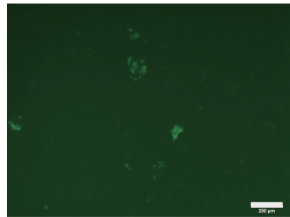

Figure S4

Figure S5

A

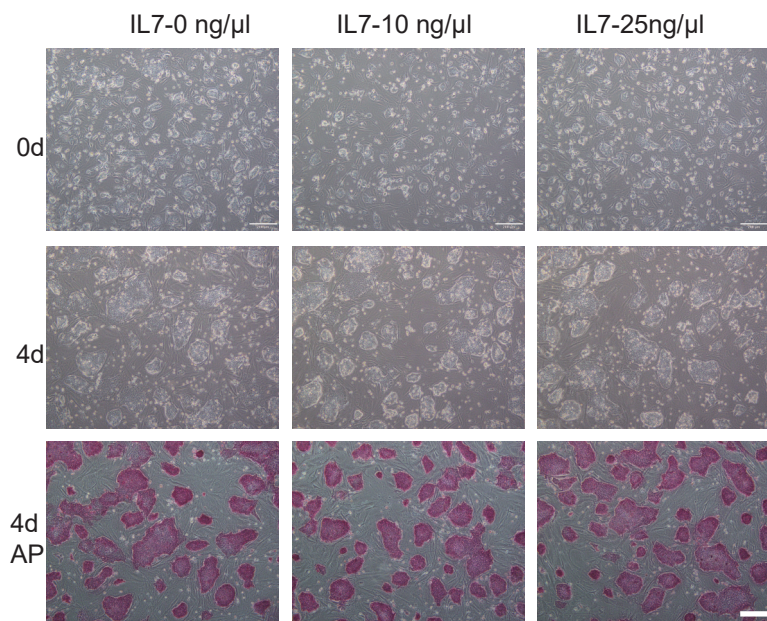

B

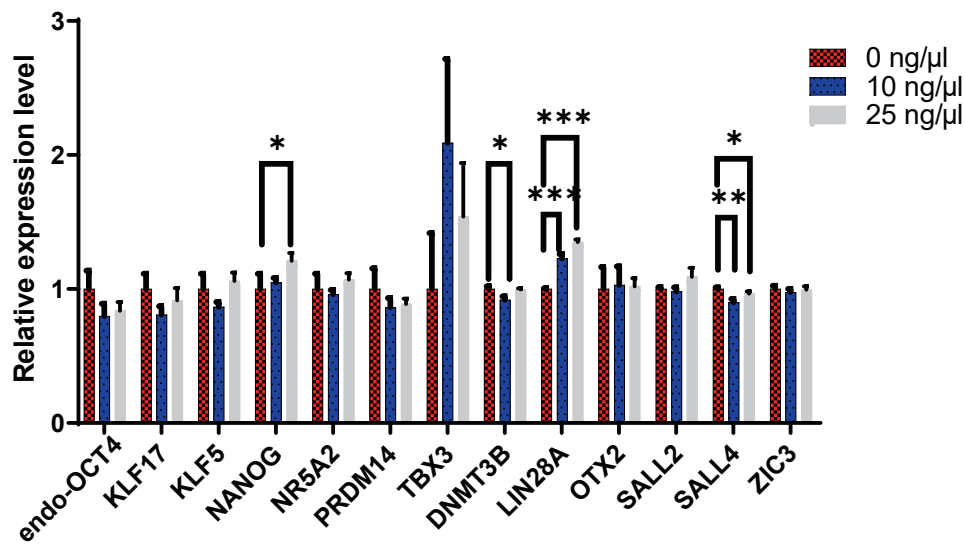

Figure S6

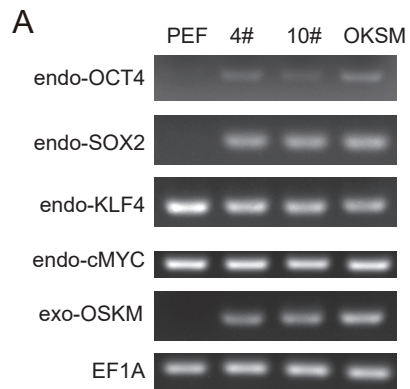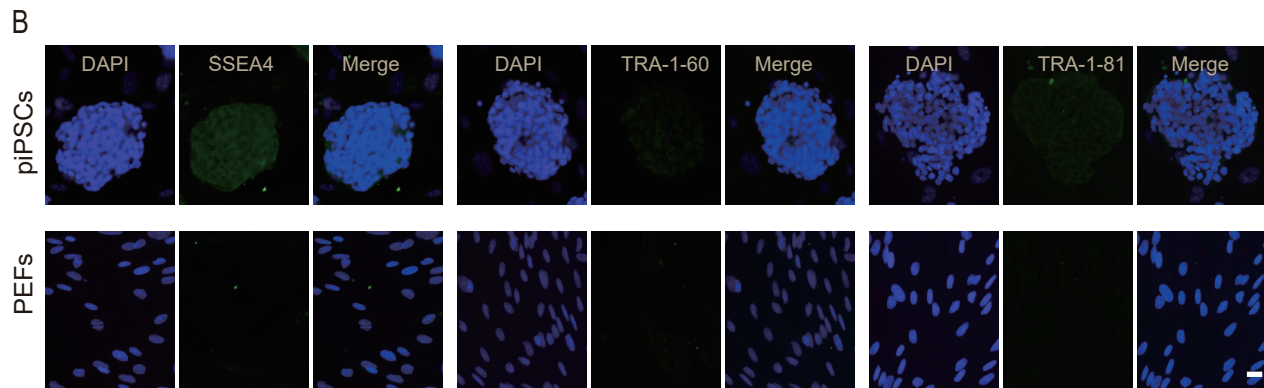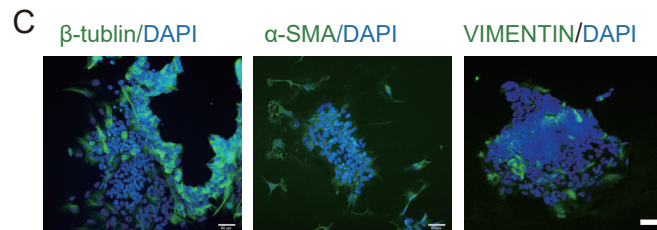

Supplement: Supplementary file 1 — Additional file 1: Figure S1. Screening for potential transcription factors related to porcine pluripotency, related to Fig. 1. (A) GO terms and KEGG pathways enriched from upregulated genes in ICM.(B) Analysis of ChIP-Seq data of H3K4me3. Marks indicate promoter regions of selected transcript factors. ChIP-Seq data of H3K4me3 in pEPSCs was obtained by Liu et al. [19]. The peaks from -4 k to -5 k upstream from the transcriptional start site of IRF-1 were peaks associated with other genes not IRF-1. (C) GFP fluorescence in PEFs transfected with reporter plasmid. Scale bar, 500 μm.(D) Distribution of red fluorescence in porcine reconstructed blastocysts by SCNT. GFP fluorescence represents successful integration of the reporter system. Red fluorescence represents the promoter’s activity. Scale bar, 100 μm.(E) qRT-PCR analysis of pluripotency-associated genes in BCL3 overexpressing cells and controls. Figure S2. Pluripotency characterization of DOX-hLIF-2i piPSCs, related to Fig. 2. (A) Immunofluorescence assay of SSEA-4, TRA-1-60, TRA-1-80. Scale bar, 20 μm. (B) EBs of DOX-hLIF-2i piPSCs obtained at day 6 of differentiation. Scale bar, 100 μm. (C) Fluorescence detection of OCT4-tdTomato in DOX-hLIF-2i piPSCs. Scale bar of the top figure, 100 μm. Scale bar of the bottom figure, 50 μm. (D) Cell morphology and AP staining of DOX-hLIF-2i piPSCs with DOX and without DOX. Scale bar, 200 μm. (E) RT-PCR analysis of endogenous expression of OCT4, SOX2, KLF4 and cMYC and exogenous OKSM. EF1A was used as internal control. 1#, 2# represent two lines of DOX-hLIF-2i piPSCs. Figure S3. The effect of IRF-1 overexpression on DOX-hLIF-2i piPSCS morphology, related to Fig. 3. (A) DAPI staining of IRF-1-overexpressing and negative control piPSCs in Fig. 3a. Scale bars from left to right, 200 μm, 50 μm. (B) RT-PCR analysis of endogenous expression of OCT4, SOX2, KLF4 and cMYC and exogenous OKSM. EF1A was used as internal control. OE: IRF-1 overexpressing piPSCs, WT: DOX-hLIF-2i piPSCs. Fi [file 13287_2020_1983_MOESM1_ESM.pdf]
